# Supplementary material for: A Comprehensive Assessment of Ultraviolet-Radiation-Induced Mutations in Flammulina filiformis Using Whole-Genome Resequencing
Source: J Fungi (Basel). 2024 Mar 20;10(3):228. doi: 10.3390/jof10030228 (PMC10971301; doi:10.3390/jof10030228)
Supplement: Supplementary file 1 [file jof-10-00228-s001.zip › Supplementary Material S8/KEGG annotation/out/64381550635650.os/KO/out_map/map04923.html]

KEGG PATHWAY: Regulation of lipolysis in adipocytes - Reference pathway


|  |  |
| --- | --- |
| **Regulation of lipolysis in adipocytes - Reference pathway** |  |

[
Pathway menu
| Organism menu
| Pathway entry
| Show description
| User data mapping
]

Lipolysis in adipocytes, the hydrolysis of triacylglycerol (TAG) to release fatty acids (FAs) and glycerol for use by other organs as energy substrates, is a unique function of white adipose tissue. Lipolysis is under tight hormonal control. During fasting, catecholamines, by binding to Gs-coupled-adrenergic receptors (-AR), activate adenylate cyclase (AC) to increase cAMP and activate protein kinase A (PKA). PKA phosphorylates target protein such as hormone-sensitive lipase (HSL) and perilipin 1 (PLIN). PLIN phosphorylation is a key event in the sequential activation of TAG hydrolysis involving adipose triglyceride lipase (ATGL), HSL, and monoglyceride lipase (MGL). During the fed state, insulin, through activation of phosphodiesterase-3B (PDE-3B), inhibits catecholamine-induced lipolysis via the degradation of cAMP.

|  |  |
| --- | --- |
| Reference pathway | 100% |
